# Long-term environmental enrichment overcomes depression, learning, and memory impairment in elderly CD-1 mice with maternal sleep deprivation exposure

**DOI:** 10.3389/fnagi.2023.1177250

**Published:** 2023-04-24

**Authors:** Yue-Ming Zhang, Ru-Meng Wei, Xue-Yan Li, Yi-Zhou Feng, Kai-Xuan Zhang, Yi-Jun Ge, Xiao-Yi Kong, Xue-Chun Liu, Gui-Hai Chen

**Affiliations:** ^1^Department of Neurology (Sleep Disorders), The Affiliated Chaohu Hospital of Anhui Medical University, Hefei, Anhui, China; ^2^Department of Neurology, The Second People’s Hospital of Hefei, Hefei Hospital Affiliated to Anhui Medical University, Hefei, Anhui, China

**Keywords:** maternal sleep deprivation, enriched environment, aging, depression, cognition, inflammation, synaptic proteins

## Abstract

Early-life stress disrupts central nervous system development and increases the risk of neuropsychiatric disorder in offspring based on rodent studies. Maternal sleep deprivation (MSD) in rodents has also been associated with depression and cognitive decline in adult offspring. However, it is not known whether these issues persist into old age. Environmental enrichment is a non-pharmacological intervention with proven benefits in improving depression and cognitive impairment; however, it is unclear whether these benefits hold for aging mice following MSD exposure. The aim of this study was to explore the effects of MSD on depression and cognition in elderly offspring CD-1 mice and to determine whether long-term environmental enrichment could alleviate these effects by improving neuroinflammation and synaptic plasticity. The offspring mice subjected to MSD were randomly assigned to either a standard environment or an enriched environment. At 18 months of age, the forced swimming and tail suspension tests were used to evaluated depression-like behaviors, and the Morris water maze test was used to evaluate cognitive function. The expression levels of hippocampal proinflammatory cytokines and synaptic plasticity-associated proteins were also measured. MSD increased depression-like behaviors and impaired cognition function in aging CD-1 offspring mice. These effects were accompanied by upregulated interleukin (IL)-1β, IL-6, and tumor necrosis factor-α expression, and downregulated brain-derived neurotrophic factor, tyrosine kinase receptor B, postsynaptic density-95, and synaptophysin expression in the hippocampus. All of these changes were reversed by long-term exposure to an enriched environment. These findings suggest that MSD exerts long-term effects on the behaviors of offspring in mice, leading to depression and cognitive impairment in older age. Importantly, long-term environmental enrichment could counteract the behavior difficulties induced by MSD through improving hippocampal proinflammatory cytokines and synaptic plasticity-associated proteins.

## 1. Introduction

During intrauterine development, the central nervous system goes through several essential developmental periods and is highly susceptible to external interference, resulting in fetal developmental reprogramming that leads to anxiety, depression, and learning and memory impairment (Babenko et al., 2015; Biney et al., 2022; Laugesen et al., 2022). Owing to pregnancy-related anatomical and hormonal alterations, especially during the third trimester, approximately two-thirds of pregnant women experience sleep dysfunction, including short sleep duration, poor sleep quality, and frequent awakenings, which have detrimental effects on both the mother and offspring (Alvarenga et al., 2013; Trzepizur et al., 2017; Silvestri and Aricò, 2019). Zhao et al. (2014) showed that offspring (21 days old) of rats subjected to maternal sleep deprivation (MSD) exhibited hippocampus-dependent learning and memory impairment based on the Morris water maze (MWM) test. Moreover, Peng et al. (2016) demonstrated that MSD in rats at different stages of pregnancy increased anxiety-and depression-like behaviors and impaired cognitive function in their young adult offspring from postnatal days 42–56. However, previous studies on the adverse effects of MSD on offspring have mainly focused on young adulthood, and it is not clear whether these effects persist in elderly offspring rodents.

The hippocampus is an important region of the limbic system associated with depression and cognitive function (Jia et al., 2023; Yoon et al., 2023). Under stimulation of various pathological factors, hippocampal immune cells release large amounts of proinflammatory cytokines that contribute to neuroinflammation, which could increase the risk of depression and cognitive decline (Wu et al., 2021; Hao et al., 2022). MSD has been found to activate the microglial cells and increase the expression levels of proinflammatory cytokines, including interleukin (IL)-1β, IL-6, and tumor necrosis factor-alpha (TNF-α), in the hippocampus, accompanied by depression and cognitive impairment (Zhao et al., 2014). Furthermore, minocycline was found to effectively reverse the learning and memory dysfunction induced by MSD through suppressing the inflammatory response (Zhao et al., 2015). Accumulating evidence indicates that hippocampal synaptic function is closely related to the underlying mechanisms of depression and cognitive impairment (Duman et al., 2016; Raven et al., 2018). The depression and cognitive dysfunction induced by MSD have been reported to be associated with neurogenesis inhibition and impaired long-term potentiation in the hippocampal CA1 region (Zhao et al., 2014; Peng et al., 2016). Moreover, the expression of synaptic plasticity-associated proteins plays an important role in maintaining structural synaptic plasticity in the hippocampus. Our previous study showed that MSD significantly downregulated brain-derived neurotrophic factor (BDNF) expression and upregulated synaptotagmin-1 (Syt-1) expression in the hippocampus, which was associated with spatial learning and memory impairment detected in the MWM (Zhang Y. M. et al., 2022). Together, these lines of evidence indicate that the hippocampal inflammatory response and synaptic dysfunction are involved in the development of depression and cognitive decline caused by MSD.

Environmental enrichment (EE), by providing larger cages containing different toys and running wheels, is well-established to stimulate the brains through sensory, physical, and intellectual surroundings. EE can strengthen the bidirectional connection between the brain and the surrounding environment to help resist different pathological factors and maintain the brain’s complete function (Mohammadian et al., 2019; Yu et al., 2020a). A large body of evidence suggests that EE can improve different types of stress-induced anxiety, depression, and cognitive impairment through a range of signaling pathways involved in inflammation, oxidative stress, mitochondrial function, insulin resistance, and synaptic plasticity (Liew et al., 2022). For example, one study showed that EE normalized the inflammation balance in the brain to reduce anxiety-and depression-like behaviors associated with infant nerve injury (Gong et al., 2018). Another study demonstrated that EE compensated for the spatial learning and memory impairment induced by social isolation in a young mice model of Alzheimer’s disease by reducing synaptic loss, inflammation, and cell apoptosis (Cao et al., 2018). Our previous studies further indicated that long-term EE could ameliorate cognitive dysfunction and synaptic proteins expression under prenatal inflammatory exposure in elderly CD-1 mice (Wu et al., 2020; Zhuang et al., 2021). However, whether long-term EE exposure improves depression and cognitive decline in elderly CD-1 mice after MSD is unclear.

Therefore, the aim of this study was to investigate the long-term adverse effects of MSD on depression and cognitive function in elderly CD-1 offspring mice. Furthermore, whether long-term EE exposure exerts beneficial effects on depression and cognitive dysfunction by altering the levels of proinflammatory cytokines and synaptic plasticity-associated proteins in the hippocampus of elderly CD-1 mice with MSD.

## 2. Materials and methods

### 2.1. Animals and treatments

**Figure 1:**
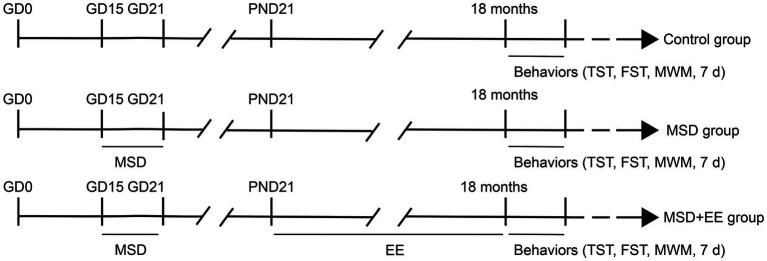
Experimental protocol. GD, gestational day; PND, postnatal day; MSD, maternal sleep deprivation; EE, environmental enrichment; TST, tail suspension test; FST, forced swimming test; MWM, Morris water maze test.

Eight-week-old female and male CD-1 mice were obtained from Beijing Vital River Laboratory Animal Company (Shanghai, China). After 2 weeks of acclimatization feeding, males and females were mated at a 1:2 ratio in a standard environment with a 12-h light/dark cycle (lights on at 8:00 AM), humidity of 50 ± 5%, and temperature of 22–25°C. Presence of a vaginal plug was observed at 8:00 AM the following day and was considered day 0 of gestation (GD 0) when detected. All mice had free access to food and water. Pregnant female mice were housed individually in cages and randomly divided into two groups: a sleep deprivation group and control group. After delivery, the offspring mice were breastfed and separated from their mothers on postnatal day 21. Offspring mice from mothers in the sleep deprivation group were divided into two groups with or without EE. Using the offspring mice as the study subjects, the analysis was based on three groups according to maternal and offspring treatment (Sixteen mice per group, including eight males and eight females): Control, MSD, MSD + EE (see Figure 1). All animal experimental procedures were performed in accordance with the guidelines for humane treatment set by the Center for Laboratory Animal Sciences and the Association of Laboratory Animal Sciences at Anhui Medical University (No. LLSC20190710).

### 2.2. Induction of sleep deprivation

A specific sleep deprivation apparatus (BW-NSD404, Shanghai Bio-will Co., Ltd.) was used to deprive the pregnant mice of sleep, as described previously (Zhang Y. M. et al., 2022). Pregnant mice were placed in the sleep deprivation apparatus daily for sleep deprivation of 6 h (12:00–18:00) during GD 15–21. The sleep deprivation apparatus was operated continuously at a rate of 0.5 m/min to ensure that the animals remained awake and the mice had free access to food and water during this time.

### 2.3. Establishment of the enriched environment

Offspring mice were provided with an enriched environment from postnatal day 21 to 18 months. Mice in the EE groups were housed in larger cages (52 × 40 × 20 cm^3^) including a variety of colorful toys, running wheels, stairs, plastic tunnels, and wooden houses, with 7–8 mice per cage. Mice not provided with EE were housed in standard cages (36 × 18 × 14 cm^3^) with three mice/cage and without any objects.

### 2.4. Tail suspension test

The tail suspension test was performed by fixing the end of the tails of the mice with tape attached to a metal hook placed 35 cm above the ground. Importantly, the tail was passed through a cylindrical plastic tube to prevent tail climbing behaviors. The entire 6-min experiment was recorded by video and the immobility time in the last 4 min was monitored and recorded by an observer blinded to the grouping.

### 2.5. Forced swimming test

The forced swimming test was performed by placing the mouse in a glass cylindrical container (28 cm in height and 18 cm in diameter) containing water (22 ± 1°C) of 15 cm depth. The entire experiment lasted for 6 min and the immobility time of the last 4 min was quantified by an observer blinded to the group. Immobility time was considered to be the time when the mouse did not exhibit any active struggling behaviors except for maintaining balance in the water. A longer immobility time in the water was considered to indicate an increase in depressive-like behavior (Ding et al., 2021).

### 2.6. Morris water maze test

The MWM test was performed in accordance with previous studies (Wu et al., 2020; Zhuang et al., 2021). The water maze apparatus consisted of a black circular pool (150 cm in diameter, 30 cm in height) containing opaque water maintained at 22°C and a target platform (10 cm in diameter, 24 cm in height). The water maze was surrounded by a white curtain with three different cue markings (triangle, square, and circle). In the spatial acquisition phase, the target platform was placed in the center of the target quadrant and located 1 cm underwater, and the position was kept fixed. Mice were randomly placed in the water from different quadrants facing the wall of the pool and allowed to explore freely for 60 s four times a day for 5 days. The mice were allowed to stay on the platform for 30 s regardless of whether they could find the target platform within 60 s. Notably, mice that did not find the target platform within 60 s were guided toward the target platform. In the probe trial phase, the target platform was removed 2 h after the end of the last training on the fifth day of the learning period. Mice were placed in the water from the quadrant opposite to the target quadrant and were allowed to explore freely for 60 s. All experimental procedures were recorded by a camera placed above the water maze. ANY-Maze (Stoeling, United States) software was used to analyze the escape latency (time spent finding the target platform), distance, and swimming velocity during the learning period, and the percentage of time and distance in the target quadrant during the memory period.

### 2.7. Enzyme-linked immunoassay

After the behavioral tests, mice were euthanized with 2% sodium pentobarbital anesthesia. The hippocampal tissue was collected from all mice at the end of the experiments, weighed, and homogenized. The supernatant was carefully collected by centrifugation for approximately 20 min (2000 × *g*). The levels of proinflammatory cytokines IL-1β, IL-6, and TNF-α were quantified using respective ELISA kits (JYM0531Mo, JYM0012Mo, and JYM0218Mo; Wuhan Colorful Gene Biotechnology Co.) according to the manufacturer’s protocol. The optical density was measured using an enzyme-labeled instrument.

### 2.8. Western blotting

Hippocampal tissues were homogenized in RIPA cell lysate (Beyotime, P0013B), centrifuged at 12,000 × *g* for 15 min, and the supernatant was collected. Each protein sample was loaded into the sodium dodecyl sulfate-polyacrylamide gel electrophoresis gel spiked wells. Electrophoresis was performed at constant pressure of 80 V for approximately 1 h. The proteins were transferred to a polyvinylidene fluoride membrane (Millipore, IPVH00010), fixed with western closure solution (5% skim milk powder), slowly shaken on a shaker for 2 h at room temperature, and then incubated with the following primary antibodies: rabbit anti-postsynaptic density-95 (PSD-95) antibody (1:2000, abcam, ab238135), rabbit anti-synaptophysin (SYN) antibody (1:1000, Bioss, bs-8845R), rabbit anti-BDNF antibody (1:1000, abcam, ab108319), and rabbit tyrosine kinase receptor B (TrkB) antibody (1:5000, abcam, ab187041). The samples were further incubated with horseradish peroxidase-conjugated goat anti-rabbit IgG secondary antibody (1:20000, Zsbio, ZB-2301) for 1.2 h. The membranes were washed with phosphate-buffered saline with Tween and the ECL luminescence kit (Thermo, 340,958) was used to detect the proteins. Finally, the intensity of the bands was analyzed by Image J software (Media Cybernetics, United States).

### 2.9. Statistical analysis

All data were statistically analyzed using GraphPad Prism 8.0 software and are expressed as mean ± standard error of the mean for each group. Differences in behavioral and biochemical outcomes were analyzed using a two-way analysis of variance (ANOVA) followed by Tukey’s *post-hoc* test. Repeated-measures ANOVA was used to analyze escape latency, distance, and swimming velocity in the learning period of the Morris water maze. Pearson’s correlation coefficient (*r*) was calculated to determine the specific relationships between BDNF, PSD-95, SYN, TrkB, IL-1β, IL-6, and TNF-α levels in the hippocampus and mouse behaviors, respectively. The differences were considered statistically significant when the *p* value was <0.05.

## 3. Results

### 3.1. EE improves depression-like behaviors induced by MSD in elderly CD-1 mice

**Figure 2:**
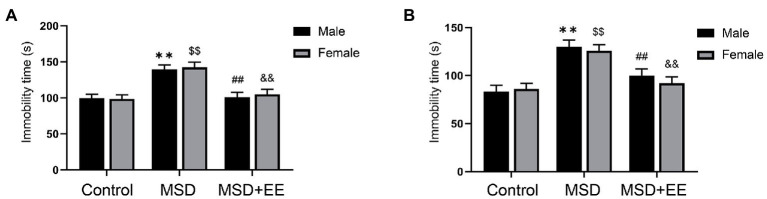
Effects of an environmental enrichment (EE) on depression-like behavior in elderly offspring mice exposed to maternal sleep deprivation (MSD). **(A)** Immobility time in the forced swimming test. **(B)** Immobility time in the tail suspension test. ^**^p < 0.01 vs. Control group in males; ^$$^*p* < 0.01 vs. Control group in females; ^##^*p* < 0.01 vs. MSD group in males; ^&&^*p* < 0.01 vs. MSD group in females.

The depression-like behavior was evaluated by the tail suspension test and forced swimming test. The immobility time was significantly different among the three groups (Control, MSD, and MSD + EE groups) during the forced swimming test [*F*_(2, 42)_ = 26.22, *p* < 0.01; Figure 2A]. *Post-hoc* analysis showed that MSD increased the immobility time when compared with that of the Control group (*p* < 0.05), which was reversed by EE (*p* < 0.05). Similarly, the immobility time was significantly different among the three groups during the tail suspension test [*F*_(2, 42)_ = 22.85, *p* < 0.01; Figure 2B]. *Post-hoc* analysis showed that the immobility time of the MSD group was significantly higher than that of the Control group (*p* < 0.05); however, the immobility time of the MSD + EE group was similar to that of the Control group. There was no sex difference in immobility time among the three groups in the forced swimming test or tail suspension test.

### 3.2. EE improves the learning and memory impairment induced by MSD in elderly CD-1 mice

**Figure 3:**
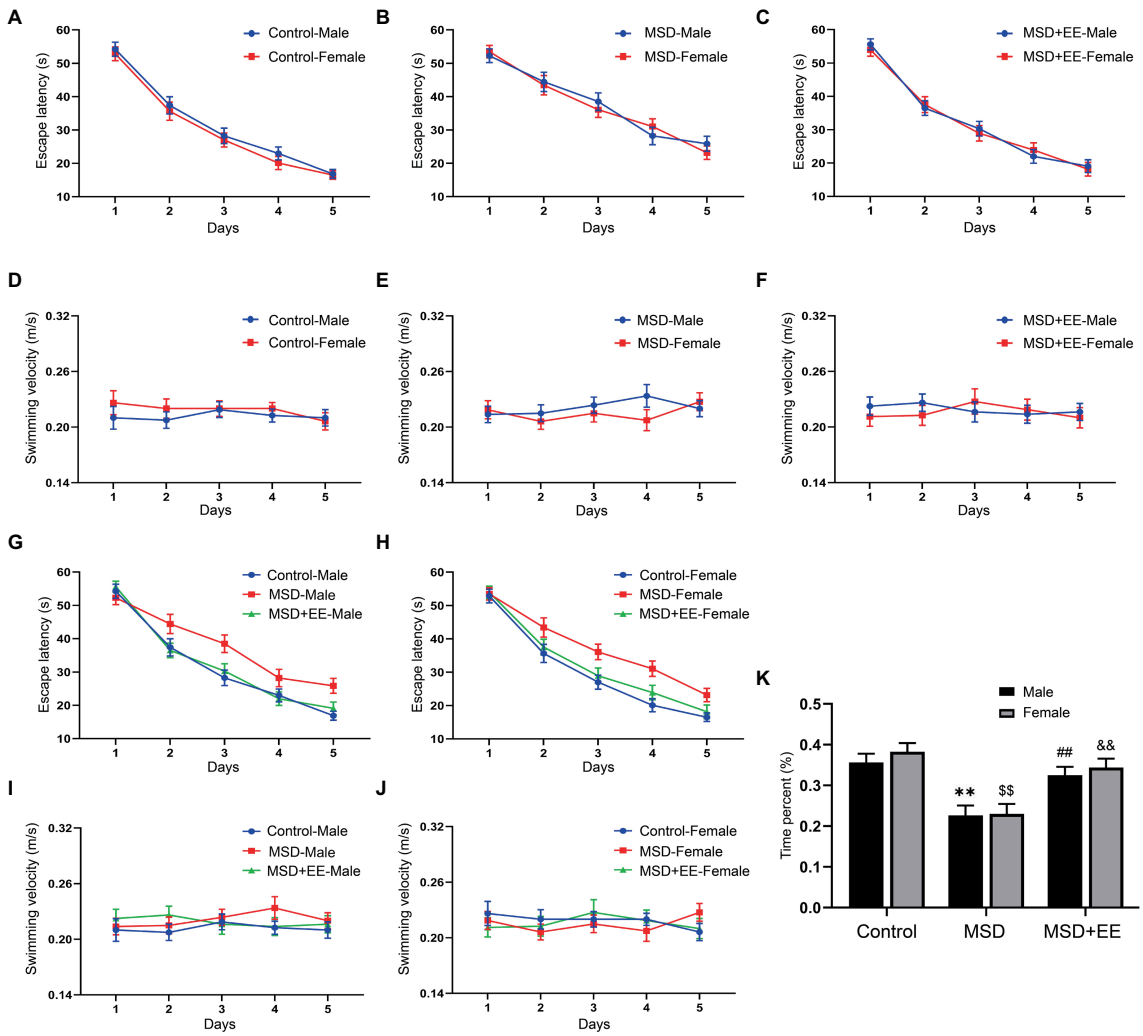
Effects of an environmental enrichment (EE) on spatial learning and memory impairment in elderly offspring mice exposed to maternal sleep deprivation (MSD). **(A–C,G,H)** Escape latency and **(D-F,I,J)** swimming velocity during the Morris water maze test. **(K)** Time spent in the target quadrant. ^**^p < 0.01 vs. Control group in males; ^$$^*p* < 0.01 vs. Control group in females; ^##^*p* < 0.01 vs. MSD group in males; ^&&^*p* < 0.01 vs. MSD group in females.

The MWM test was used to evaluate hippocampus-dependent learning and memory function in elderly CD-1 mice following MSD exposure. In the learning phase, with the increase of training days, the escape latency and distance for each group to locate the hidden platform gradually decreased (Figures 3A–C,G,H; Supplementary Figures S1A–E). Controlling for treatment, there were no significant differences found in escape latency and distance among the three groups (Figures 3A–C; Supplementary Figures S1A–C). However, when the analysis was controlled for sex, the escape latency and distance were significantly different among the three groups (escape latency: males: *F*_(2,21)_ = 6.66, *p* < 0.01; females: *F*_(2,21)_ = 7.90, *p* < 0.01; Figures 3G,H; distance: males: *F*_(2,21)_ = 8.19, *p* < 0.01; females: *F*_(2,21)_ = 7.21, *p* < 0.01; Supplementary Figures S1D,E). *Post-hoc* analysis revealed that the MSD group spent longer and moved a greater distance locating the hidden platform than the Control group (*Ps* < 0.05). Both the time and distance were decreased in the MSD + EE group compared with those of the MSD group (*Ps* < 0.05). There was no difference in the escape latency and distance between the Control group and MSD + EE group. There were no statistical differences in swimming velocity between groups when controlling for sex or treatment (Figures 3D–F,I,J).

In the memory phase, the time and distance in the target quadrant were significantly different among the three groups (time: *F*_(2,42)_ = 21.73, *p* < 0.01; Figure 3K; distance: *F*_(2,42)_ = 18.77, *p* < 0.01; Supplementary Figure S1F). Furthermore, the MSD group had a shorter time and distance in the target quadrant than the Control group or MSD + EE group (*Ps* < 0.05). There was no significant difference in the time and distance between the Control and MSD + EE groups.

### 3.3. EE improves proinflammatory cytokines induced by MSD in hippocampus of elderly CD-1 mice

**Figure 4:**
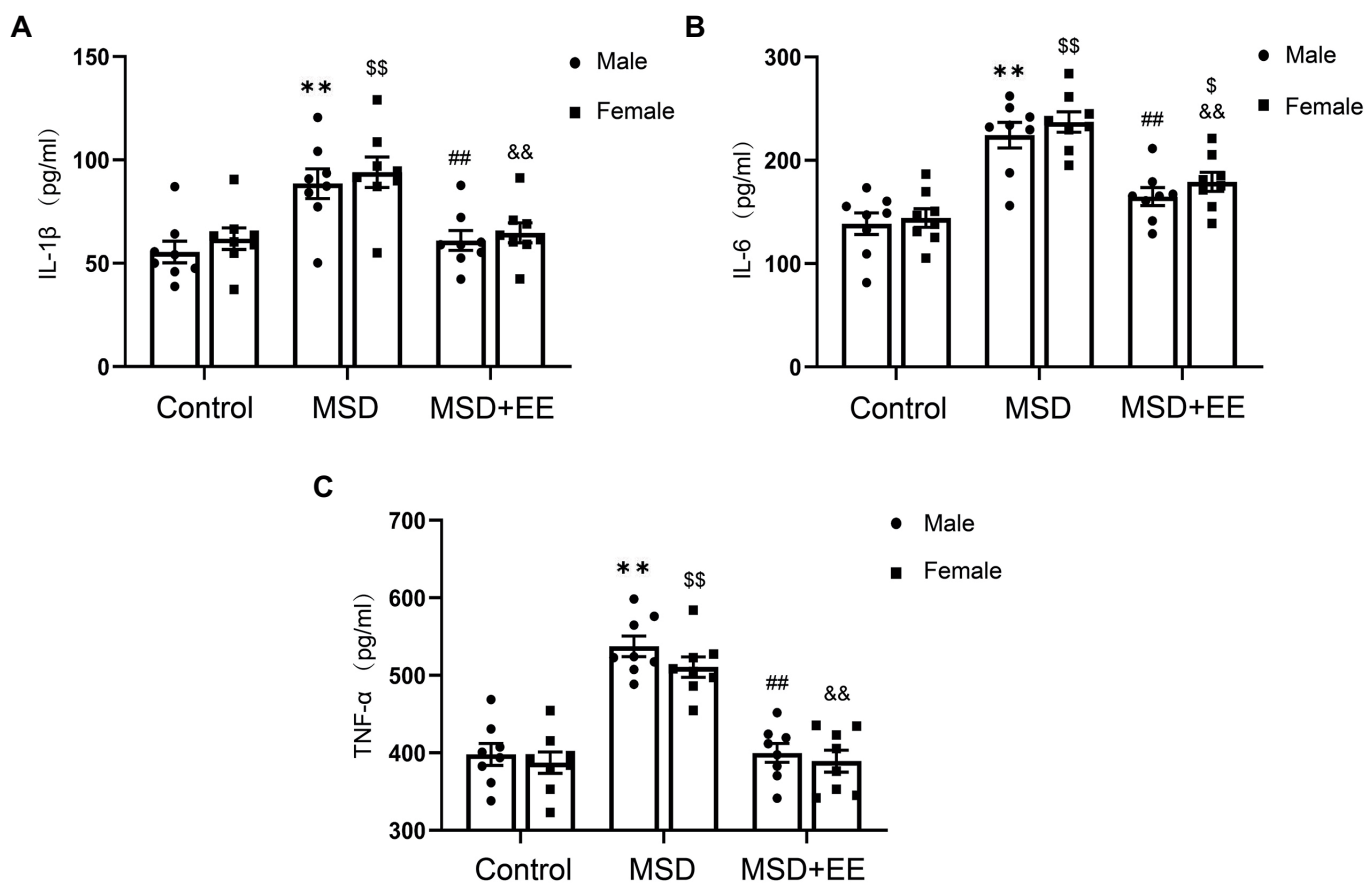
Effects of an environmental enrichment (EE) on inflammation mediators in the hippocampus of elderly offspring mice exposed to maternal sleep deprivation (MSD). **(A)** Expression level of IL-1β in the hippocampus. **(B)** Expression level of IL-6 in the hippocampus. **(C)** Expression level of TNF-α in the hippocampus. ^**^p < 0.01 vs. Control group in males; ^$^*p* < 0.05, ^$$^*p* < 0.01 vs. Control group in females; ^##^*p* < 0.01 vs. MSD group in males; ^&&^*p* < 0.01 vs. MSD group in females.

The expression levels of hippocampal proinflammatory cytokines, including IL-1β, IL-6, and TNF-α, were significantly different among the three groups (IL-1β: *F*_(2,42)_ = 18.23, *p* < 0.01; IL-6: *F*_(2,42)_ = 40.83, *p* < 0.01; TNF-α: *F*_(2,42)_ = 61.89, *p* < 0.01; Figures 4A–C). *Post-hoc* analysis showed that the levels of these proinflammatory cytokines in the hippocampus of the MSD group were significantly higher than those of the Control group (*Ps* < 0.05), whereas these levels were significantly decreased in the MSD + EE group compared with those of the MSD group (*Ps* < 0.05).

### 3.4. EE improves the MSD-induced decrease in synaptic plasticity-associated proteins in hippocampus of elderly CD-1 mice

**Figure 5:**
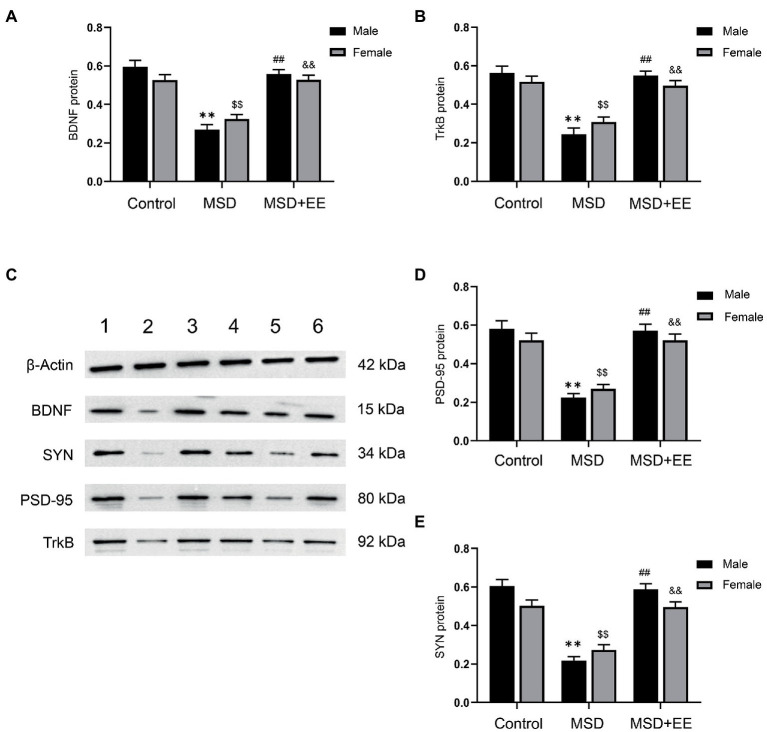
Effects of an environmental enrichment (EE) on synaptic plasticity-associated proteins in the hippocampus of elderly offspring mice exposed to maternal sleep deprivation (MSD). **(A)** Expression level of BDNF in the hippocampus. **(B)** Expression level of TrkB in the hippocampus. **(C)** Representative western blotting of BDNF, TrkB, PSD-95, and SYN proteins in the hippocampus: band 1, Control group-male; band 2, MSD-group male; band 3, MSD + EE group-male; band 4, Control group-female; band 5, MSD group-female; band 6, MSD + EE group-female. **(D)** Expression level of PSD-95 in the hippocampus. **(E)** Expression level of SYN in the hippocampus. ^**^p < 0.01 vs. Control group in males; ^$$^*p* < 0.01 vs. Control group in females; ^##^*p* < 0.01 vs. MSD group in males; ^&&^*p* < 0.01 vs. MSD group in females.

The western blotting results showed that the protein expression levels of BDNF, TrkB, PSD-95, and SYN in the hippocampus were significantly different among the three groups (BDNF: *F*_(2,30)_ = 65.29, *p* < 0.01; TrkB: *F*_(2,30)_ = 52.88, *p* < 0.01; PSD-95: *F*_(2,30)_ = 60.92, *p* < 0.01; SYN: *F*_(2,42)_ = 75.96, *p* < 0.01; Figures 5A–E). *Post-hoc* analysis showed that the expression levels of BDNF, TrkB, PSD-95, and SYN in the hippocampus of the MSD group were all significantly lower than those of the Control group (*Ps* < 0.05). However, EE reversed this effect, with increased expression levels of BDNF, TrkB, PSD-95, and SYN in the MSD + EE group compared with those of the MSD group (*Ps* < 0.05).

### 3.5. Correlations between depression/cognitive performance and the expression levels of proinflammatory cytokines/synaptic plasticity associated proteins

The immobility time of the tail suspension test and forced swimming test (as a proxy of depression-like behavior) was positively correlated with the levels of proinflammatory cytokines (IL-1β, IL-6, and TNF-α) and was negatively correlated with the levels of synaptic plasticity-associated proteins (BDNF, TrkB, PSD-95, and SYN), as shown in Table 1.

**Table 1:** Correlations between performance in the depression/cognition-related tasks and hippocampal proinflammatory cytokines.

Tasks	Indexes	Groups	Proinflammatory cytokines	
			IL-1β	IL-6	TNF-α
Tail suspension test	Immobility time	Control-male	0.776 (0.024)*	0.546 (0.161)	0.763 (0.028)*
		MSD-male	0.930 (0.001)**	0.824 (0.012)*	0.809 (0.015)*
		MSD + EE-male	0.889 (0.003)**	0.899 (0.002)**	0.935 (0.001)**
		Control-female	0.804 (0.016)*	0.445 (0.269)	0.470 (0.240)
		MSD-female	0.856 (0.007)**	0.922 (0.001)**	0.896 (0.003)**
		MSD + EE-female	0.829 (0.011)*	0.784 (0.021)*	0.759 (0.029)*
Forced swimming test	Immobility time	Control-male	0.505 (0.202)	0.718 (0.045)*	0.701 (0.053)
		MSD-male	0.846 (0.008)**	0.769 (0.026)*	0.807 (0.015)*
		MSD + EE-male	0.910 (0.002)**	0.950 (0.000)**	0.852 (0.007)**
		Control-female	0.827 (0.011)*	0.559 (0.150)	0.574 (0.137)
		MSD-female	0.905 (0.002)**	0.762 (0.028)*	0.893 (0.003)**
		MSD + EE-female	0.852 (0.007)**	0.825 (0.012)*	0.760 (0.029)*
Morris water maze test	Escape latency	Control-male	0.741 (0.036)*	0.575 (0.136)	0.750 (0.032)*
		MSD-male	0.872 (0.005)**	0.814 (0.014)*	0.947 (0.000)**
		MSD + EE-male	0.899 (0.002)**	0.859 (0.006)**	0.771 (0.025)*
		Control-female	0.560 (0.149)	0.789 (0.020)*	0.711 (0.048)*
		MSD-female	0.758 (0.029)*	0.836 (0.010)**	0.780 (0.022)*
		MSD + EE-female	0.751 (0.032)*	0.807 (0.015)*	0.834 (0.010)*
	Percentage of time swam	Control-male	−0.709 (0.049)*	−0.819 (0.013)*	−0.826 (0.011)*
		MSD-male	−0.753 (0.031)*	−0.909 (0.002)**	−0.806 (0.016)*
		MSD + EE-male	−0.748 (0.033)*	−0.827 (0.011)*	−0.856 (0.007)**
		Control-female	−0.758 (0.029)*	−0.743 (0.035)*	−0.740 (0.036)*
		MSD-female	−0.844 (0.008)**	−0.837 (0.009)**	−0.834 (0.010)*
		MSD + EE-female	−0.755 (0.030)*	−0.812 (0.014)*	−0.905 (0.002)**

*Denotes a significant correlation (^*^P < 0.05; ^**^P < 0.01).

MSD, maternal sleep deprivation; EE, environmental enrichment; IL, interleukin; TNF, tumor necrosis factor.

With respect to cognitive tasks, the average escape latency and distance in the learning phase were negatively correlated with the levels of synaptic plasticity-associated proteins, whereas the percent time and distance in the memory phase were positively correlated with the levels of synaptic plasticity-associated proteins. The average escape latency and distance in the learning phase were positively correlated with the levels of proinflammatory cytokines, whereas the percent time and distance of the memory phase were negatively correlated with the levels of proinflammatory cytokines (Table 2; Supplementary Tables S1, S2).

**Table 2:** Correlations between depression/cognition-related tasks and hippocampal synaptic plasticity-associated proteins.

Tasks	Indexes	Groups	Synaptic proteins	
			BDNF	TrkB	PSD-95	SYN
Tail suspension test	immobility time	Control-male	−0.823 (0.044)*	−0.888 (0.018)*	−0.668 (0.147)	−0.755 (0.083)
		MSD-male	−0.820 (0.046)*	−0.758 (0.081)	−0.874 (0.023)*	−0.887 (0.019)*
		MSD + EE-male	−0.938 (0.006)**	−0.963 (0.002)**	−0.919 (0.010)**	−0.939 (0.005)**
		Control-female	−0.806 (0.053)	−0.905 (0.013)*	−0.826 (0.043)*	−0.790 (0.062)
		MSD-female	−0.943 (0.005)**	−0.926 (0.008)**	−0.843 (0.035)*	−0.921 (0.009)**
		MSD + EE-female	−0.908 (0.012)*	−0.910 (0.012)*	−0.854 (0.031)*	−0.812 (0.050)*
Forced swimming test	Immobility time	Control-male	−0.970 (0.001)**	−0.939 (0.006)**	−0.902 (0.014)*	−0.935 (0.006)**
		MSD-male	−0.911 (0.012)*	−0.831 (0.040)*	−0.878 (0.021)*	−0.865 (0.026)*
		MSD + EE-male	−0.915 (0.011)*	−0.992 (0.000)**	−0.839 (0.037)*	−0.810 (0.051)
		Control-female	−0.968 (0.002)**	−0.946 (0.004)**	−0.984 (0.000)**	−0.962 (0.002)**
		MSD-female	−0.873 (0.023)*	−0.848 (0.033)*	−0.976 (0.001)**	−0.924 (0.008)**
		MSD + EE-female	−0.874 (0.023)*	−0.873 (0.023)*	−0.851 (0.032)*	−0.794 (0.059)
Morris water maze test	Escape latency	Control-male	−0.334 (0.518)	−0.492 (0.322)	−0.179 (0.734)	−0.219 (0.677)
		MSD-male	−0.838 (0.037)*	−0.848 (0.033)*	−0.918 (0.010)**	−0.891 (0.017)*
		MSD + EE-male	−0.965 (0.002)**	−0.994 (0.000)**	−0.876 (0.022)*	−0.901 (0.014)*
		Control-female	−0.866 (0.026)*	−0.707 (0.116)	−0.885 (0.019)*	−0.874 (0.023)*
		MSD-female	−0.985 (0.000)**	−0.967 (0.002)**	−0.892 (0.017)*	−0.963 (0.002)**
		MSD + EE-female	−0.904 (0.013)*	−0.863 (0.027)*	−0.954 (0.003)**	−0.933 (0.007)**
	Percentage of time swam	Control-male	0.926 (0.008)**	0.950 (0.004)**	0.762 (0.078)	0.838 (0.037)*
		MSD-male	0.909 (0.012)*	0.965 (0.002)**	0.875 (0.022)*	0.847 (0.033)*
		MSD + EE-male	0.943 (0.005)**	0.843 (0.035)*	0.879 (0.021)*	0.979 (0.001)**
		Control-female	0.913 (0.011)*	0.826 (0.043)*	0.948 (0.004)**	0.914 (0.011)*
		MSD-female	0.914 (0.011)*	0.865 (0.026)*	0.956 (0.003)**	0.919 (0.010)**
		MSD + EE-female	0.962 (0.002)**	0.927 (0.008)**	0.919 (0.010)**	0.905 (0.013)*

*Denotes significant correlation (^*^P < 0.05; ^**^P < 0.01).

MSD, maternal sleep deprivation; EE, environmental enrichment; BDNF, brain-derived neurotrophic factor; TrkB, tyrosine kinase receptor B; PSD-95, postsynaptic density-95; SYN, synaptophysin.

## 4. Discussion

In this study, we performed a series of behavioral tests (forced swimming test, tail suspension test, and MWM test) to evaluate the long-term detrimental effects of MSD on depression and cognitive function, and further measured the levels of hippocampal inflammatory cytokines and synaptic plasticity-associated proteins to identify the beneficial effects of long-term EE from weaning in aging CD-1 mice exposed to MSD. Our results demonstrated that MSD resulted in depression and cognitive impairment in elderly CD-1 mice, whereas the EE intervention improved depression-like behaviors and cognitive impairment through limiting the hippocampal inflammation response and synaptic dysfunction.

### 4.1. EE improves MSD-induced depression and cognitive impairment in elderly CD-1 mice

Accumulating evidence suggests that perinatal insults and stress could have long-term effects on brain tissue and structure in later life, thereby increasing the risk of neuropsychiatric disorders. Aged offspring rats exposed to cocaine and/or nicotine during GD 8–20 showed anxiety-and depression-like behaviors in the elevated plus maze and sucrose preference test (Sobrian et al., 2003). Prenatal long-term exposure to electromagnetic radiation exerted adverse effects on the cognitive function of elderly rats as assessed in the MWM test (Hong et al., 2020). In line with these previous findings, in the present study, we found that MSD increased the level of depression in aging offspring mice, as evidenced by increased immobility time in the MSD group compared to the Control group during the forced swimming and tail suspension tests. The MWM test is a traditional and effective tool to measure hippocampus-dependent learning and memory function (Bromley-Brits et al., 2011). We also found that MSD increased the escape latency and distance in the learning phase and decreased the target quadrant time and distance in the memory phase of the MWM, implying impaired spatial learning and memory in elderly offspring after MSD exposure.

It is well known that hippocampal neurogenesis, dendritic arborization, synaptic plasticity, and neuronal connections are continuously modified by experiences and learning. EE has been reported to improve impaired brain function by providing more sensory, cognitive, and social stimuli to reverse depression and cognitive impairment (Vrinda et al., 2017; Akbari et al., 2020; Wang et al., 2020). A previous study showed that EE effectively improved depression-like behaviors induced by chronic unpredictable mild stress as assessed in forced swimming and sucrose preference tests (Shen et al., 2019). In humans, enriching the community environment by providing social activities, spiritual consolation services, and health care services was found to slow age-related cognitive decline (Yu and Wei, 2021). The present study demonstrated that long-term EE exposure improved MSD-induced depression and cognitive impairment in elderly mice. This result is consistent with the findings of our previous studies that EE alleviated the age-related cognitive impairment induced by prenatal inflammatory exposure (Ni et al., 2022; Zhang Z. Z. et al., 2022). Collectively, these findings suggest that MSD results in behavioral difficulties in offspring that can persist into old age, which could be reversed by long-term EE. This should highlight the importance of paying more attention to the mental health of older people who have experienced perinatal stress, and if possible, try to help pregnant mothers avoid stress events and subsequent psychological trauma.

Additionally, the MSD and EE had no effect on the swimming velocity in the elderly mice during the MWM test, suggesting that the beneficial effect of EE on spatial learning and memory was not related to changes in motor activity.

### 4.2. EE improves MSD-induced inflammatory response and synaptic plasticity

Previous studies found that hippocampal microglial cells activated by different stress could transform their morphology and increase their motility to activate a series of inflammatory signaling pathways, leading to the upregulation of proinflammatory cytokines in the hippocampus (Karve et al., 2016; Yang et al., 2021). Moreover, upregulated inflammatory cytokines in the hippocampus are closely related to depression and cognitive impairment. For example, long-term corticosterone treatment significantly increased the mRNA levels of IL-1β, IL-6, and TNF-α in the hippocampus and hypothalamus, which in turn increased the anxiety-and depression-like behaviors in mice (Chabry et al., 2015). The elevated IL-1 and IL-10 expression induced by lipopolysaccharide (LPS) impaired learning and memory assessed in the Morris water maze test (Keymoradzadeh et al., 2020). Furthermore, the progression of cognitive impairment associated with Alzheimer’s disease involves the accumulation of proinflammatory cytokines in the hippocampus (Ates et al., 2020; Chen C. et al., 2022). Consistently, the present study showed that MSD increased the expression levels of the proinflammatory factors IL-1β, IL-6, and TNF-α in the hippocampus, which may have contributed to the depression and cognitive dysfunction in the elderly offspring mice.

Activated microglial cells can be divided into the M1 classical phenotype and M2 replacement phenotype. The activated M1 microglial cells contribute to the secretion of proinflammatory cytokines and nervous system dysfunction, whereas M2 microglial cells can secrete neurotrophic factors to alleviate the inflammation associated with central nervous system damage (Liao et al., 2012; Kobayashi et al., 2013). A recent study suggested that anti-inflammatory treatment reversed MSD-induced spatial learning and memory deficits in offspring mice, which was related to decreased levels of inflammatory cytokines and inhibition of the M1-biased microglial response (Zhao et al., 2015). Our present results showed that long-term EE effectively decreased the expression levels of IL-1β, IL-6, and TNF-α, and improved depression and cognitive deficits in the MSD + EE group compared with those of the MSD group. The association between the brain’s immune function and living environment has been well studied. EE increased the markers of the M2 microglial phenotype and reduced the production of inflammatory cytokines induced by chronic stress (Gu et al., 2021). Additionally, EE could enhance autophagy function to inhibit unpredictable chronic stress-increased inflammation activation and proinflammatory cytokines production (Xu et al., 2022). In view of the above evidence, it is reasonable to hypothesize that EE alleviated MSD-induced proinflammatory cytokines by inhibiting microglial activation and increasing autophagy function.

Hippocampal synaptic plasticity is considered to be a mechanism of depression and cognitive dysfunction (Xu et al., 2021; Tartt et al., 2022). BDNF is a well-studied neurotrophic factor that plays an important role in the regulation of synaptic plasticity (Spies et al., 2023). BDNF binds to its receptor TrkB to control cell proliferation, differentiation, survival, dendritic sprouting, and synaptic transmission (Numakawa et al., 2010). Chronic restraint-stressed mice showed increased depression-like behavior and downregulation of the BDNF/TrkB signaling pathway. Inversely, upregulation of the BDNF/TrkB signaling pathway exerted anti-depression effects on chronic restraint-stress-induced depression (Wu et al., 2023). Downregulation of the BDNF/TrkB signaling pathway was also found in mice with LPS-induced learning and memory dysfunction (Li et al., 2017). The present results showed that MSD decreased the protein expression levels of BDNF and TrkB in the elderly offspring, which further supports that decreased expression of BDNF and TrkB may be a potential cause of depression and cognitive impairment. A previous study showed that the downregulation of BDNF expression induced by maternal sevoflurane exposure was reversed by EE through increasing the level of histone acetylation (Yu et al., 2020b). Consistently, we found that EE reversed the MSD-induced downregulation of the BDNF/TrkB signaling pathway to improve depression and cognitive impairment. These results are also in accordance with a previous study showing that EE improved autism spectrum disorder-associated cognitive impairment by activating the BDNF/TrkB signaling pathway (Chen Y. S. et al., 2022).

SYN and PSD-95 are synaptic proteins that promote synaptic plasticity, and their brain expression levels are reduced in cases of depression and cognitive impairment, which could be reversed by exposure to an enriched environment (Sifonios et al., 2009; Hong et al., 2020). Similarly, we found that EE reversed MSD-decreased SYN and PSD-95 expression in the hippocampus of elderly offspring mice, implying that EE exerts anti-depression and cognitive protection by improving the expression of synaptic proteins.

### 4.3. Correlations between the markers of inflammation/synaptic plasticity and depression/cognitive impairment

Emerging evidence indicates that the expression levels of proinflammatory cytokines and synaptic plasticity-associated proteins are strongly associated with depression and cognitive dysfunction. Previous studies suggested that the expression levels of BDNF and inflammatory cytokines in the peripheral blood are related to the degree of depression and cognitive impairment of patients (Gelle et al., 2021; He et al., 2021). Our recent studies showed that upregulated expression of proinflammatory cytokines and downregulated expression of synaptic proteins are associated with age-related cognitive impairment (Ni et al., 2022; Zhang Z. Z. et al., 2022). Similarly, we found significant correlations between the expression levels of proinflammatory cytokines or synaptic plasticity-associated proteins and indicators of the tail suspension test, forced swimming test, and MWM test. Our findings therefore suggest that the beneficial effects of EE on the behavioral dysfunction induced by MSD may be associated with decreased production of proinflammatory cytokines and increased production of synaptic plasticity-associated proteins in the hippocampus.

Previous studies have suggested that there are sex differences in the effects of early life stress on behavioral phenotype of offspring rodents. For example, one study showed that cognitive decline and synaptic dysfunction were observed in male offspring mice suffered from maternal separation, but not in female offspring mice (Talani et al., 2023). Other study found that prenatal restraint stress significantly increased anxiety-like behavior in male offspring mice and attenuated it in female offspring mice (Zuena et al., 2008). In the present study, MSD and EE had similar effects on behaviors, inflammatory response, and synaptic function between male and female aging offspring CD-1 mice, which is inconsistent with previous study showed that MSD has a sex-dependent effect on the sexual behavior in young offspring Wistar-Hannover rats (Alvarenga et al., 2013). The inconsistent results may be attributed to the time, duration, frequency of MSD, conditions of MSD, strain, as well as individual characteristics such as age.

There are some limitations of this study. First, we only demonstrated a link between behavior dysfunction, the inflammatory response, and synaptic plasticity-associated proteins at the phenomenal level, and did not demonstrate that improvement in inflammation and synaptic plasticity-associated proteins could ameliorate depression and cognitive dysfunction directly. Second, we did not set up an additional Control+EE group because the beneficial effects of EE on cognition and BDNF expression have been reported previously (Lores-Arnaiz et al., 2006). Finally, we did not detect alterations of proinflammatory cytokines and synaptic plasticity-associated proteins in other regions associated with depression and cognition, such as the prefrontal cortex and hypothalamus (Bao and Swaab, 2018; Chakraborty et al., 2019).

## 5. Conclusion

In summary, the present study showed the long-term adverse effects induced by MSD in elderly offspring mice, and confirmed that MSD resulted in depression and spatial learning and memory impairment, accompanied by increased levels of proinflammatory cytokines and decreased levels of synaptic plasticity-associated proteins in the hippocampus of mice. Moreover, long-term EE could ameliorate MSD-induced depression and spatial learning and memory impairment by reversing alterations in proinflammatory cytokines and synaptic plasticity-associated proteins.

## Data availability statement

The original contributions presented in the study are included in the article Supplementary material, further inquiries can be directed to the corresponding author/s.

## Ethics statement

All animal experiments complied with the guidelines for humane treatment established by the Association of Laboratory Animal Sciences and the Center for Laboratory Animal Sciences of the Anhui Medical University (NO. LLSC20190710).

## Author contributions

Y-MZ and R-MW designed the study, performed the behavioral tests, and drafted the manuscript. X-YL, Y-ZF, and K-XZ were responsible for the western blotting and enzyme-linked immunoassay. Y-JG and X-YK analyzed the data and constructed the graphs. X-CL and G-HC revised the manuscript and were responsible for the completeness and accuracy of the data. All authors read and approved the final manuscript.

## Funding

This work was financially supported by the National Natural Science Foundation of China (grant number: 81671316), College of the Natural Science Foundation of Anhui Province (2022AH050759), and 2022 Key Research and Development Plan of Anhui Province (2022e07020029).

## Conflict of interest

The authors declare that the research was conducted in the absence of any commercial or financial relationships that could be construed as a potential conflict of interest.The reviewer FW declared a shared parent affiliation with the authors to the handling editor at the time of review.

## Publisher’s note

All claims expressed in this article are solely those of the authors and do not necessarily represent those of their affiliated organizations, or those of the publisher, the editors and the reviewers. Any product that may be evaluated in this article, or claim that may be made by its manufacturer, is not guaranteed or endorsed by the publisher.
