# Supplementary material for: Long-term environmental enrichment overcomes depression, learning, and memory impairment in elderly CD-1 mice with maternal sleep deprivation exposure
Source: Front Aging Neurosci. 2023 Apr 24;15:1177250. doi: 10.3389/fnagi.2023.1177250 (PMC10164971; doi:10.3389/fnagi.2023.1177250)
Supplement: Supplementary file 1 [file Table_1.DOCX]

Supplementary Material

Long-Term Environmental Enrichment Overcomes Depression, Learning, and Memory Impairment in Elderly CD-1 Mice With Maternal Sleep Deprivation Exposure

Yue-Ming Zhang, Ru-Meng Wei, Xue-Yan Li, Yi-Zhou Feng, Kai-Xuan Zhang, Yi-Jun Ge, Xiao-Yi Kong, Xue-Chun Liu^*^, Gui-Hai Chen^*^

*** Correspondence:** Gui-Hai Chen: [doctorcgh@163.com](mailto:doctorcgh@163.com); Xue-Chun Liu: [xuechun0724@hotmail.com](mailto:xuechun0724@hotmail.com)

**1 Supplementary Figures and Tables**

**1.1 Supplementary Figures**


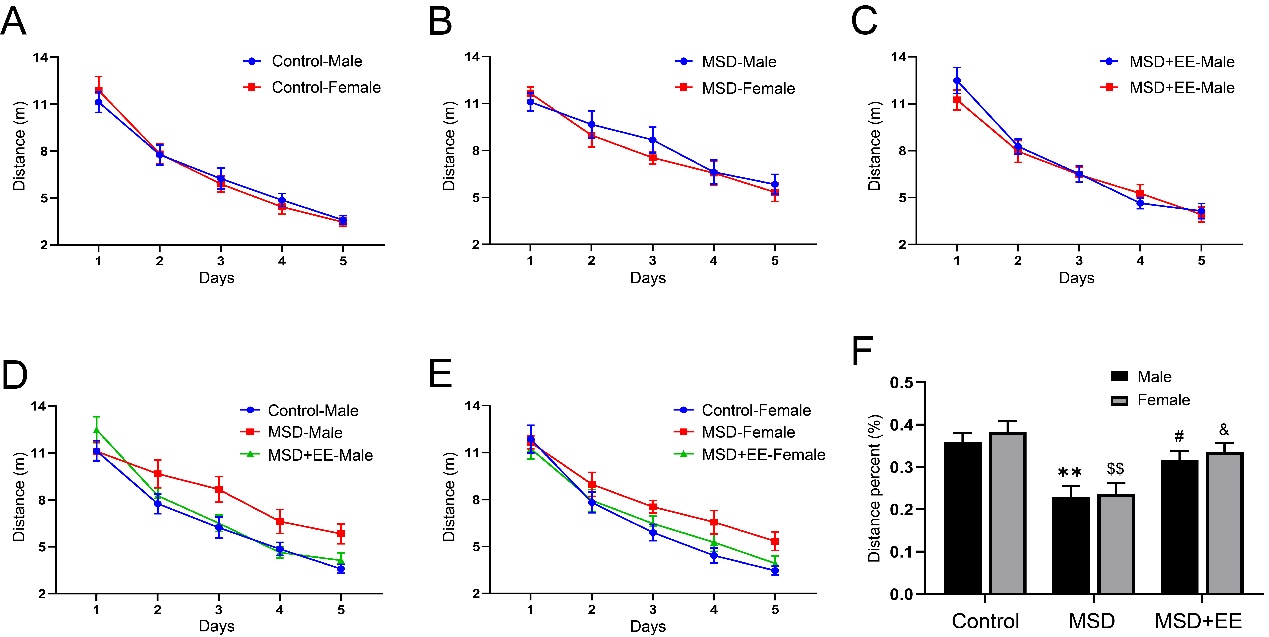


**Supplemental Figure 1.** Effects of environmental enrichment (EE) on spatial learning and memory impairment in elderly offspring mice exposed to maternal sleep deprivation (MSD). **(A–E)** Distance travelled during the Morris water maze test. **(F)** Distance spent in the target quadrant. ^**^*P* < 0.01 vs. Control group in males; ^$$^*P* < 0.01 vs. Control group in females; ^#^*P* < 0.05 vs. MSD group in males; ^&^*P* < 0.05 vs. MSD group in females.

**1.2 Supplementary Tables**

**Supplementary Table 1** Correlations between the cognition-related tasks in the Morris water maze test and hippocampal proinflammatory cytokines.

| Tasks | Indexes | Groups | Proinflammatory cytokines | |  |
| --- | --- | --- | --- | --- | --- |
|  |  |  | IL-1β | IL-6 | TNF-α |
| Morris water maze test | Distance swam | Control-male | 0.636 (0.090) | 0.733 (0.039)* | 0.816 (0.014)* |
|  |  | MSD-male | 0.864 (0.006)** | 0.847 (0.008)** | 0.910 (0.002)** |
|  |  | MSD+EE-male  Control-female  MSD-female  MSD+EE-female | 0.807 (0.015)*  0.764 (0.027)*  0.854 (0.007)**  0.826 (0.011)* | 0.787 (0.021)*  0.769 (0.026)*  0.829 (0.011)*  0.829 (0.011)* | 0.746 (0.033)*  0.667 (0.071)  0.805 (0.016)*  0.808 (0.015)* |
|  | Percentage of distance swam | Control-male | -0.626 (0.097) | -0.799 (0.017)* | -0.786 (0.021)* |
|  |  | MSD-male | -0.784 (0.021)* | -0.938 (0.001)** | -0.799 (0.017)* |
|  |  | MSD+EE-male  Control-female  MSD-female  MSD+EE-female | -0.781 (0.022)*  -0.756 (0.030)*  -0.830 (0.011)*  -0.722 (0.043)* | -0.864 (0.006)**  -0.777 (0.023)*  -0.813 (0.014)*  -0.769 (0.026)* | -0.803 (0.016)*  -0.728 (0.040)*  -0.828 (0.011)*  -0.880 (0.004)** |

*Denotes significant correlation (^*^*P* < 0.05; ^**^*P* < 0.01)

Abbreviations: MSD, maternal sleep deprivation; EE, environmental enrichment; IL, interleukin; TNF, tumor necrosis factor.

**Supplementary Table 2** Correlations between the cognition-related tasks in the Morris water maze test and hippocampal synaptic plasticity-associated proteins.

| Tasks | Indexes | Groups | Synaptic proteins | |  |  |
| --- | --- | --- | --- | --- | --- | --- |
|  |  |  | BDNF | TrkB | PSD-95 | SYN |
| Morris water maze test | Distance swam | Control-male | -0.439 (0.383) | -0.593 (0.215) | -0.585 (0.223) | -0.506 (0.305) |
|  |  | MSD-male | -0.871 (0.024)* | -0.868 (0.025)* | -0.872 (0.023)* | -0.817 (0.047)* |
|  |  | MSD+EE-male  Control-female  MSD-female  MSD+EE-female | -0.912 (0.011)*  -0.916 (0.010)*  -0.967 (0.002)**  -0.927 (0.008)** | -0.913 (0.011)*  -0.795 (0.059)  -0.918 (0.010)**  -0.910 (0.012)* | -0.869 (0.024)*  -0.909 (0.012)*  -0.908 (0.012)*  -0.928 (0.008)** | -0.812 (0.050)*  -0.927 (0.008)**  -0.951 (0.004)**  -0.879 (0.021)* |
|  | Percentage of distance swam | Control-male | 0.981 (0.001)** | 0.982 (0.001)** | 0.898 (0.015)* | 0.942 (0.005)** |
|  |  | MSD-male | 0.917 (0.010)* | 0.983 (0.000)** | 0.901 (0.014)* | 0.880 (0.021)* |
|  |  | MSD+EE-male  Control-female  MSD-female  MSD+EE-female | 0.882 (0.020)*  0.921 (0.009)**  0.941 (0.005)**  0.895 (0.016)* | 0.940 (0.005)**  0.822 (0.044)*  0.905 (0.013)*  0.847 (0.033)* | 0.976 (0.001)**  0.938 (0.006)**  0.912 (0.011)*  0.846 (0.034)* | 0.822 (0.045)*  0.927 (0.008)**  0.914 (0.011)*  0.849 (0.033)* |

*Denotes significant correlation (^*^*P* < 0.05; ^**^*P* < 0.01)

Abbreviations: MSD, maternal sleep deprivation; EE, environmental enrichment; BDNF, brain-derived neurotrophic factor; TrkB, tyrosine kinase receptor B; PSD-95, postsynaptic density-95; SYN, synaptophysin.
